# Machine Learning for Efficient Prediction of Protein Redox Potential: The Flavoproteins Case

**DOI:** 10.1021/acs.jcim.2c00858

**Published:** 2022-09-20

**Authors:** Bruno Giovanni Galuzzi, Antonio Mirarchi, Edoardo Luca Viganò, Luca De Gioia, Chiara Damiani, Federica Arrigoni

**Affiliations:** † Department of Biotechnology and Biosciences, University of Milano-Bicocca, Piazza della Scienza 2, 20126 Milan, Italy; ‡ Istituto di Ricerche Farmacologiche Mario Negri, Via Mario Negri 2, 20156 Milan, Italy; § SYSBIO Centre of Systems Biology/ISBE.IT, Piazza della Scienza 2, 20126, Milan, Italy

## Abstract

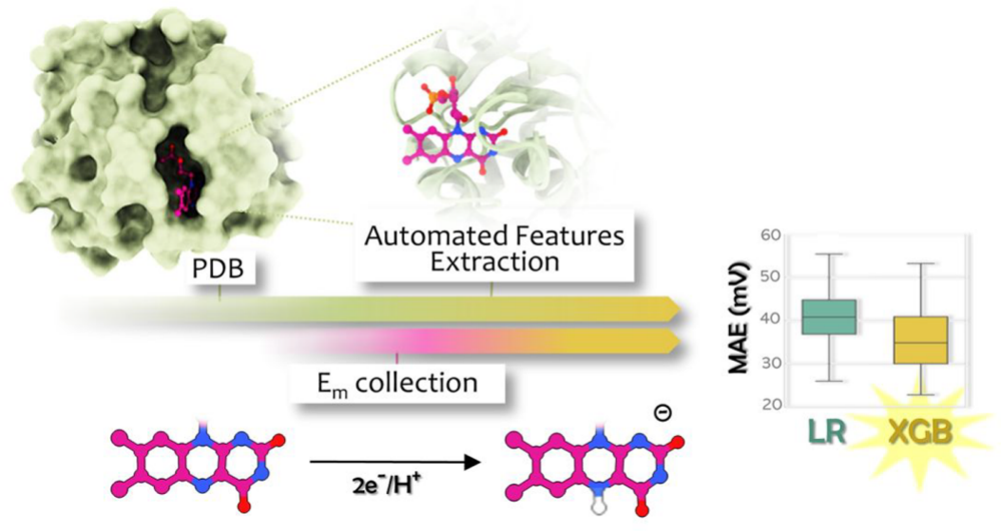

Determining the redox potentials of protein cofactors and how they are influenced by their molecular neighborhoods is essential for basic research and many biotechnological applications, from biosensors and biocatalysis to bioremediation and bioelectronics. The laborious determination of redox potential with current experimental technologies pushes forward the need for computational approaches that can reliably predict it. Although current computational approaches based on quantum and molecular mechanics are accurate, their large computational costs hinder their usage. In this work, we explored the possibility of using more efficient QSPR models based on machine learning (ML) for the prediction of protein redox potential, as an alternative to classical approaches. As a proof of concept, we focused on flavoproteins, one of the most important families of enzymes directly involved in redox processes. To train and test different ML models, we retrieved a dataset of flavoproteins with a known midpoint redox potential (*E*_m_) and 3D structure. The features of interest, accounting for both short- and long-range effects of the protein matrix on the flavin cofactor, have been automatically extracted from each protein PDB file. Our best ML model (XGB) has a performance error below 1 kcal/mol (∼36 mV), comparing favorably to more sophisticated computational approaches. We also provided indications on the features that mostly affect the *E*_m_ value, and when possible, we rationalized them on the basis of previous studies.

## Introduction

The qualitative and quantitative evaluations of the relationships between the redox properties of protein cofactors and their molecular environments are key areas of study for both basic research and technological applications.^1−4^ Structure–property relationships in molecular systems are not always experimentally accessible (e.g., in research projects aimed to design proteins with tailored redox properties). Consequently, computational approaches that allow for a reliable and fast prediction of protein redox potential are very important to complement and enrich the data obtained from “wet” experiments.

**Scheme 1:**
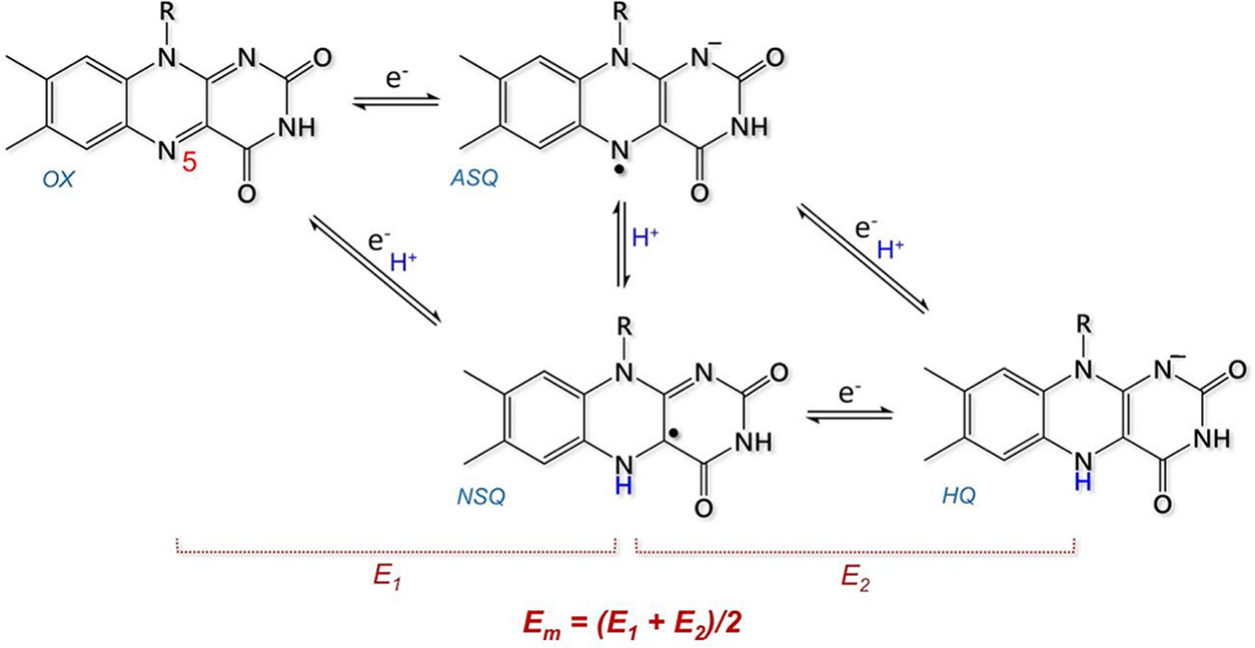
Redox and Protonation States Accessible to the Isoalloxazine Ring in Flavoproteins: Quinone (OX), Semiquinone (either as anionic, ASQ, or neutral, NSQ, species), and Hydroquinone (HQ)

Among the very large number of enzymes directly involved in redox processes, flavoproteins represent one of the most important families, both for the high number of known flavoproteins and for the large variety of redox reactions catalyzed by these enzymes,^5,6^ due to their ability to catalyze either one or two-electron transfer reactions. In fact, flavins can go through three relevant redox forms (Scheme 1): quinone (OX), semiquinone (either as anionic, ASQ, or neutral, NSQ, species), and hydroquinone (HQ).

In addition, flavoproteins are very promising systems for biotechnological applications, where the availability of enzymatic molecular systems with tailored redox behavior is crucial. Current and prospective biotechnological applications include biosensors,^7^ biocatalysis,^7,8^ bioremediation,^9^ and bioelectronics.^10^

Due to the relevance and versatility of flavoproteins, several systematic studies have been carried out with the aim of disclosing key structural determinants affecting the redox properties of the flavin cofactor.^11−13^ As an example, structural and functional studies on flavodoxins have established that electrostatic interactions are a dominant factor affecting the SQ/HQ equilibrium. In particular, since the flavin hydroquinone in flavodoxins is not protonated at N1,^14^ the isoalloxazine moiety is anionic, and it is expected to generate substantial repulsions in the negatively charged protein environment commonly observed in flavodoxins.^15,16^ Indeed, mutations in *D. vulgaris* flavodoxin have revealed a strong correlation of the NSQ/HQ potential with the number of negatively charged groups in the neighborhood of the flavin,^16^ confirming that the flavin mononucleotide cofactor bound to flavodoxins is more difficult to convert to the fully reduced form than free FMN. Investigation of wild type and mutated flavodoxins from *D. vulgaris*(16−19) and *C. beijerinckii*(15) showed that unfavorable aromatic stacking interactions can also play critical roles in tuning the redox potential. Several other studies have also highlighted and disclosed the roles of specific hydrogen bonds and electrostatic, hydrophobic, and π–π stacking interactions, as well as conformational changes of the tricyclic ring or its environment on the flavin reduction potential.^20−23^ However, the quantitative prediction of the effects of these interactions and features on the redox potential of flavoproteins is extremely difficult to predict because the contribution of these effects is expected to scale in a nonlinear fashion and is therefore particularly difficult to quantify only on the ground of structural analysis. The redox potentials of proteins can be computed using *ab initio*, semiempirical, or classical methods,^24−27^ some of which were tested and used to predict the redox potential of flavoproteins.^28,29^ Truhlar and collaborators reported a series of seminal density functional theory investigations about lumiflavin in different solvents and with different substituents, which were used as benchmarks for subsequent quantum mechanics/molecular mechanics (QM/MM) studies aimed at investigating the redox properties of small flavoproteins.^30^ However, even though QM and QM/MM studies allow one to estimate the flavin reduction potential with an average error of only 10–20 mV, the massive and systematic application of QM and QM/MM methods in virtual screening protocols is still hindered by the large computational cost of such calculations.^31−33^ In parallel to QM and QM/MM studies, approaches based on a molecular mechanics description of flavoproteins have been reported. Specifically, Sattelle and Sutcliffe^34^ carried out a thermodynamic integration (TI) study on a series of natural and engineered flavodoxins, differing for one amino acid in the cofactor environment. Also, in this case, results were very encouraging, with an average difference between calculated and measured redox potential of 20–100 mV. However, the computational cost of a TI investigation is also quite large, and it does not yet allow one to investigate many possible flavoprotein variants differing for one or more amino acids in a systematic way.

Machine learning (ML) might be a promising approach for a computationally efficient prediction of protein redox potential, since it can be generally used to assist and derive quantitative structure–property relationships (QSPR) for chemical systems.^35−38^ Once the computationally demanding step of model training and testing is completed, a ML model can indeed be employed to quickly predict the redox potential of any flavoprotein. Recently, ML techniques have been extensively applied to predict, or rationalize, quantitative relationships between molecular structures and properties, showing how these methods can nicely and successfully complement *ab initio* or semiempirical approaches.^39−53^ However, to the best of our knowledge, ML has not been used yet for the prediction of protein redox potential. Prompted by these considerations, we propose a ML-based QSPR pipeline that can be used within a high-throughput framework to predict the redox potential of flavoproteins using only their 3D structure as input.

To build a labeled dataset, suitable for the training and testing of ML-based QSPR models, we scanned the scientific literature searching for information on the redox potential associated with available 3D structures. From each identified molecular structure, we automatically extracted 246 features that may influence the redox potential. To take into account both local and global factors, we considered the physicochemical properties for both the whole protein and a portion of it. We compared the performances of different ML regression models, namely, linear regression, support vector regression, Gaussian process regression, k-nearest neighborhood, random forest, and extreme gradient boosting, as well as different methods of feature extraction. In addition, we analyzed the importance of each feature for the accuracy of the prediction, and when possible, we rationalized our results on the basis of previous investigations.

## Results

### Flavoproteins Dataset

The first step to train and test each regression model was the reconstruction of a labeled dataset. To this aim, we identified a dataset of flavoproteins for which both the 3D structure and the midpoint redox potential (*E*_m_) were known. In this study, we focused only on *E*_m_, which is the average redox potential between *E*_1_ and *E*_2_ (Scheme 1), and not on the one-electron redox processes *E*_1_ and *E*_2_, to increase as much as possible the number of entries in the dataset (which is a crucial parameter for robust training and prediction). In fact, for several flavoproteins of the dataset only *E*_m_ was available. Indeed, *E*_1_ and *E*_2_ are hard to measure, for example, because the flavoprotein reacts as a two-electron redox species, or the cofactor is characterized by crossed redox potential.^54−56^

**Figure 1:**
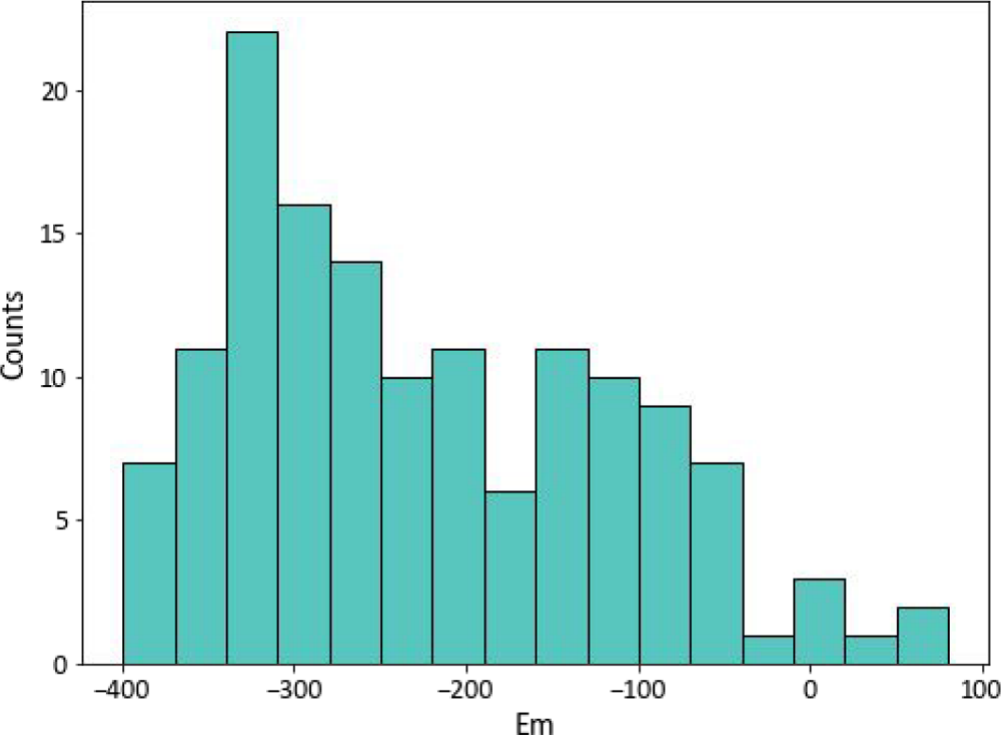
Histogram of distribution of redox potential in the flavoprotein dataset used in this study.

We retrieved most flavoproteins from the Flavoprotein Database (http://flavoproteindbwebdev-theflavoproteindatabase.webplatformsunpublished.umich.edu/) and some others by applying a systematic literature search strategy (see Methods for further details). We considered only flavoproteins with noncovalently bound flavin(s) and including a single cofactor (FAD or FMN). We obtained a set of 141 flavoproteins. The dataset covers a wide range of redox potentials (see Figure 1, μ = −223 mV, σ = 109 mV, max = 71 mV, min = −399 mV) and significant structural variation, at different levels. First, it is composed of various families of flavoproteins, such as oxidoreductases and electron-transporting proteins, with different overall architectures (Supporting Information File 2). Second, for each protein (when possible), variants from different species are included that have the same fold but different sequences (Supporting Information File 2) (note: the inclusion of proteins from different organisms in the same dataset should not affect the performance of the model, since we do not expect that there are species–specific variables that may affect flavin redox potential). Third, mutants with available 3D structures and redox potentials were also included, allowing one to tune the model sensitivity toward subtle structural changes.

The method used to acquire all the 3D structures collected in the dataset is X-ray diffraction. The resolution ranges between 0.78 Å (3W5H) and 3.3 Å (2B76), with a mean value of 2 Å and a standard deviation of 0.47 Å. When multiple PDB files were available for the same flavoprotein, we considered the one with the highest resolution.

For each protein in the dataset, we extracted several molecular descriptors that may affect the *E*_m_ value. We focused on descriptors that take into account *exclusively* the properties of the protein amino acids, thus excluding those features regarding the chemical interaction pattern of the cofactor with the protein residues (such as the presence of H-bond(s) between a specific flavin atom and its surroundings). In this way, the model will be as general as possible, potentially applicable to any type of protein regardless of the chemical nature of the cofactor considered. The cofactor coordinates are used, when necessary, just as reference points to extract the features of interest, on the basis of geometric considerations (*vide infra*).

**Figure 2:**
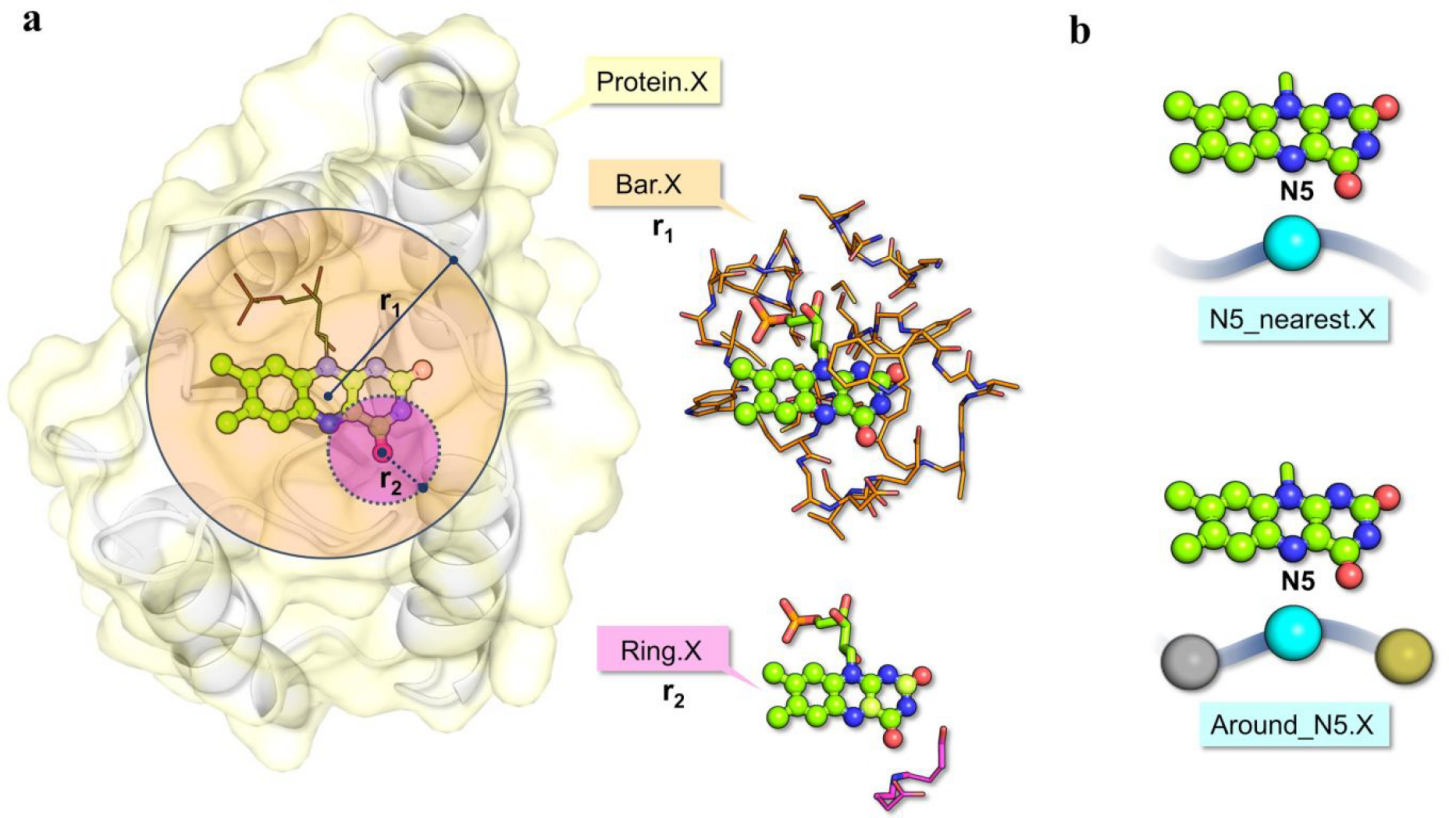
Examples of features for flavin surrounding description. (a) Features related to physicochemical properties of protein portions of decreasing size: from the entire protein (Protein.X descriptors, in yellow) to a sphere defined by r_1_ (Bar.X descriptors, in orange) to smaller spheres defined by r_2_ (Ring.X descriptors, in pink). (b) Features describing the nature and property of residue(s) found in the proximity of N5 (N5_nearest.X and Around.N5.X descriptors).

The descriptors that we used provide information about the following:

(i) Electronic, steric, and overall physicochemical properties (165 features), including countings (e.g., of charged, hydrophobic, polar, and apolar residues or of specific residue types) and sums of physicochemical quantities (e.g., volume, flexibility, hydrophobicity) taken from ref (57) for residues belonging to either the entire protein or a spheric portion of the protein surrounding the flavin cofactor to include from long-range effects to strictly local effects. The definition of the size of the flavin environment to be considered, and of an adequate cutoff radius, is of central relevance and nontrivial. For this reason, we followed different strategies to define a cutoff distance from the isoalloxazine moiety (Figure 2a). This set of 165 features results from the union set of 55 features repeatedly extracted according to the three following strategies:
  - Considering the entire protein. This subset of features is labeled as “Protein.X” where “X” describes the feature (for example, “Protein.ResPolar” refers to the number of polar residues in the entire protein chain).
  - Considering a sphere of radius *r*_1_, centered in the barycenter of the isoalloxazine ring of the flavin. We labeled the features of this subset as “Bar.X” (for example, “Bar.nNats in side chain” refers to the number of N atoms contained in the side chain of all the residues found within a *r*_1_ distance from isoalloxazine barycenter).
  - Considering spheres of radius *r*_2_ centered on each atom of the isoalloxazine ring. These descriptors are labeled as “Ring.X” (for example, “Ring.Steric hindrance” refers to the steric hindrance of residues found at a *r*_2_ distance from the N1 atom of the isoalloxazine ring). In this case, three additional features were extracted, counting the number of nitrogen, oxygen, and carbon atoms within *r*_2_ (labeled Nitrogen_Around, Oxygen_Around, and Carbon_Around, respectively). In this way, the accuracies of the chemical descriptions of molecular groups found in close proximity to the flavin (thus likely interacting with it) are increased.
(ii) Properties of the amino acids located in proximity to the N5 atom of the isoalloxazine ring (28 features) because it is known that the nature of the residue(s) interacting with N5 can strongly affect flavin redox potential (Figure 2b).^20,58^ Flavin N5 changes its protonation state along with the double reduction, and protonation can occur at the semiquinone or quinone state according to the N5 environment, thus altering *E*_m_. The same 28 features were calculated for the residue nearest to N5, labeled “N5_nearest_X”, and for the same residue plus the two adjacent ones in the amino acid sequence, labeled “Around_N5.X” (for example, Around_N5.Hydrophobicity describes the sum of the hydrophobicity, as defined in ref (57) of the three residues that are found in proximity to N5). Note: In the case of application to other protein families, with a different cofactor, these features may be neglected, or alternatively, N5 may be replaced by another atom or group of atoms that, for instance, change protonation state upon reduction. These descriptors and the ones described in (i) should also implicitly capture the essential physicochemical properties and steric features of the flavin binding site.
(iii) Composition, transition, and distribution of amino acid attributes along the amino acid sequence (21 features). Introduced by Dubchak and collaborators,^59^ these features describe the global attribute of residue “types” in a protein (such as hydrophobicity, secondary structure, and solvent accessibility) and their relative positions along the sequence. This class of descriptors was calculated using the integrated “CTD” function (composition, transition, distribution) by PyBioMed library.^60^
(iv) pH value at which the measure of *E*_m_ was carried out (when available).

In total, 246 molecular descriptors were calculated for each flavoprotein. The values of the descriptors depend on the choice of the radii *r*_1_ and *r*_2_. To assess the robustness of the regression models as a function of the derived features, we considered different combinations of *r*_1_ and *r*_2_. In particular, we scanned *r*_1_ = 8, 9, ...,16 Å, and *r*_2_ = 3, 4, 5, 6 Å, for a total of 36 different configurations. Hence, we obtained 36 different final datasets of dimension 141 × 247 (features plus *E*_m_). Note that we tested different combinations of bar radius *r*_1_ and ring radius *r*_2_, scanning values greater than the worst PDB resolution in the dataset.

### XGB Outperforms Other Estimators

For each of the obtained 36 datasets, we compared the prediction performance of six different estimators, namely, linear regression (LR), support vector regression (SVR), k-nearest neighborhood (KNR), Gaussian process regression (GPR), random forest (RF), and extreme gradient boosting (XGB) (Methods). All tested estimators underwent a 5-fold cross validation on the training data (80%) to find the best combination of hyperparameters, with a grid search strategy (details in Methods). Once the best hyperparameters were obtained, the estimator was retrained with the optimal hyperparameters set on the entire training set, while the prediction performance was evaluated on the subset of unseen data (20%). We repeated the overall procedure 10 times to evaluate the performance variability in terms of mean and standard deviation.

**Figure 3:**
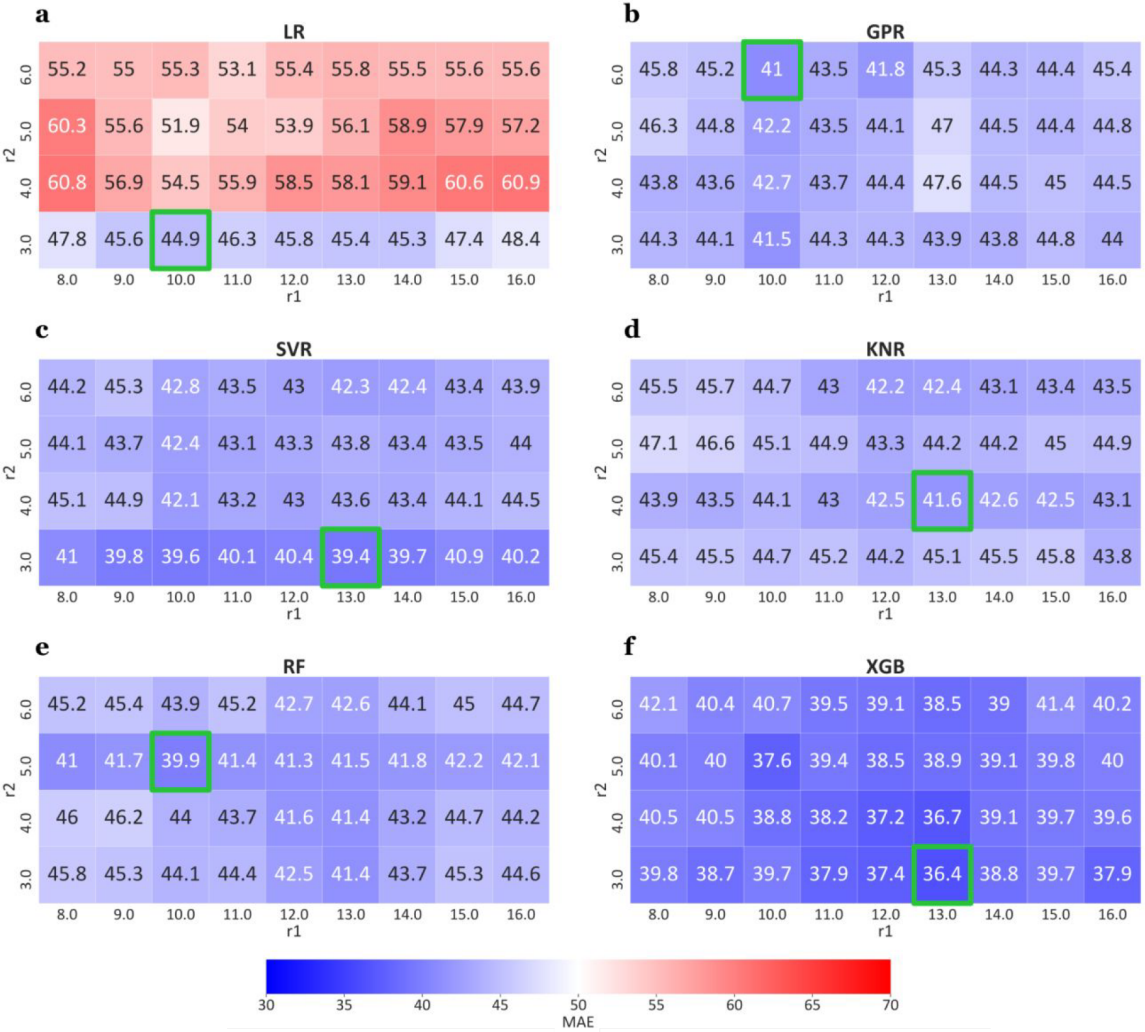
Comparison of MAE values (mV) using LR (a), GPR (b), SVR(c), KNR(d), RF(e), and XGB(f) for different radii, *r*_1_ and *r*_2_ (in Å). For each estimator, the lowest MAE is highlighted by a green box.

In Figure 3, we report for each estimator the mean absolute error (MAE) as a function of the radii *r*_1_ and *r*_2_. Each value represents the mean across the 10 repetitions. All the ML models, except for the LR estimator (Figure 3a), reach a MAE value lower than 42 mV for at least one combination of radii. In fact, LR clearly shows a worse performance compared to the other models with the MAE values ranging from 44.9 mV (*r*_1_ = 10 Å, *r*_2_ = 3 Å) to 60.9 mV (*r*_1_ = 16 Å, *r*_2_ = 4 Å). This result indicates that a simple linear relationship is not sufficient to well describe the relationship between input and output. On the contrary, the XGB model (Figure 3f) always outperforms the other models (Figure 3a–e), for any choice of the radii *r*_1_ and *r*_2_, with the MAE values ranging from 36.4 mV (*r*_1_ = 13 A°, *r*_2_ = 3 Å) to 41.4 mV (*r*_1_ = 15 Å, *r*_2_ = 6 Å). Most estimators tend to show better performances when a low value for *r*_1_ and a medium value for *r*_2_ are used. No model achieved its best performance for *r*_2_ < 10 Å or *r*_2_ > 13 Å. Worth noting is that two models (XGB and SVR, Figure 3f and c) achieved the best performance for the same configuration, i.e., *r*_1_ = 13 Å and *r*_2_ = 3 Å.

In view of the results of the comparison of different configurations of *r*_1_ and *r*_2_, we considered for each estimator its specific best radii *r*_1_ and *r*_2_ configuration (e.g., *r*_1_ = 10 Å and *r*_2_ = 3 Å for LR), and we analyzed in more detail the performances of the models. The mean values of MAE, root mean squared error (RMSE), square correlation coefficient (R2), and Spearman correlation (SC) for the best radii configurations and their standard deviations are reported in Table 1. Regarding the mean values, the XGB model achieved better predictive performance than other methods, with the lowest MAE and RMSE values and the highest R2 and SC. In more detail, the XGB model achieved a MAE of 36.36 mV, a RMSE of 51.99 mV, a R2 of 0.75, and a SC of 0.87. LR displayed the worst performance for all the metrics.

**Table 1:** Comparison of Mean and Standard Deviation of MAE (mV), RMSE (mV), R2, and SC

Model	r_1_	r_2_	MAE	RMSE	R2	SC
LR	10	3	44.87 ± 6.70	61.18 ± 10.65	0.66 ± 0.14	0.81 ± 0.09
GPR	10	6	40.96 ± 8.44	57.98 ± 12.28	0.69 ± 0.13	0.85 ± 0.06
RF	10	5	39.87 ± 6.86	57.65 ± 10.28	0.70 ± 0.10	0.86 ± 0.06
KNR	13	4	41.60 ± 6.16	59.57 ± 9.64	0.68 ± 0.12	0.85 ± 0.04
SVR	13	3	39.39 ± 6.95	56.10 ± 10.5	0.71 ± 0.12	0.85 ± 0.06
XGB	13	3	36.36 ± 6.13	51.99 ± 9.87	0.75 ± 0.11	0.87 ± 0.06

In summary, the best performance is obtained with XGB, followed by SVR.

Given the non-negligible value of the standard deviations observed for all the metrics, we investigated whether the differences in the performances obtained by XGB and the other models are statistically significant. To this aim, we performed a pairwise statistical analysis based on the Mann–Whitney U rank test. We rejected the null hypothesis if *p* < 0.05. All the obtained *p*-values of the statistical analysis are reported in the Supporting Information File S2. Generally, we observed that XGB performs better than GPR, KNR, and LR for all the performance metrics and, in most cases, better than SVR and RF. There are some cases in which the distribution of XGB is not statistically different from those of RF and SVR. For example, regarding the MAE values, we observed that the performance of XGB is not significantly different from SVR (*p* = 0.054). However, taken together, our results indicate that XGB generally outperforms all the other models.

### SHAP Values Explain the Feature Relevances

Besides the possibility of using ML models to predict the redox potential of new flavoproteins, it is relevant to exploit them to investigate the features that have greater impacts on modulation of the output. Therefore, once it is ascertained that XGB produces the best ML model, we retrained it using the entire dataset (reinserting the test set) with *r*_1_ = 3 Å and *r*_2_ = 13 Å, and we computed SHAP values (Methods) to study the impact that each feature has on the predicted *E*_m_ value.

**Figure 4:**
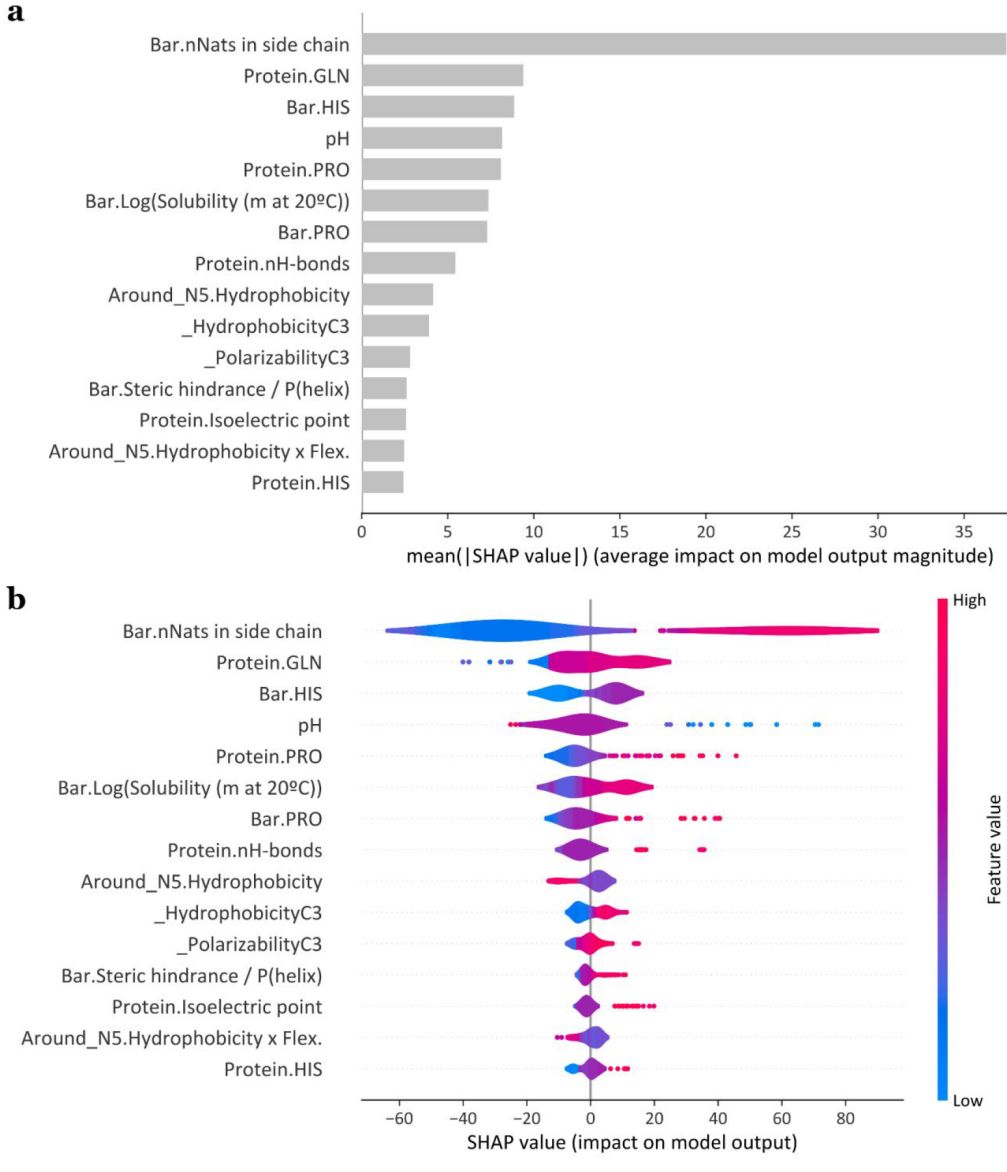
(a) SHAP summary plot for XGB trained on the entire flavoprotein dataset, with *r*_1_ = 3 Å and *r*_2_ = 13 Å. The *x*-axis stands for the average absolute SHAP values, and the *y*-axis has the first 15 features ranked in descending order. (b) SHAP violin plot for the same model in (a). The *x*-axis displays the distribution of SHAP values, and the color represents the directional impact of the features (higher values of the feature are marked in red, whereas lower values are marked in blue).

In Figure 4, we reported the SHAP summary plot (a) and the violin plot (b) for the best ML model (i.e., XGB). In both plots, features are ranked in descending order (average absolute SHAP values). In Figure 4a, the horizontal location shows whether that feature influences or not the model prediction. Figure 4b displays a violin plot of the distribution of the SHAP values. Positive SHAP values indicate a positive impact on the prediction, and negative SHAP values indicate a negative impact. The color represents the directional impact of the feature (higher values of the feature are marked in red, whereas lower values are marked in blue). As it can be observed from the barplot and violin plots, the number of N (nitrogen) atoms in the molecular neighborhood calculated with respect to the barycenter (“bar.nNats in side chain”) appears to have a particularly high impact. Indeed, it strongly correlates with the experimentally observed midpoint redox potential values (Supporting Information File S1, Figure S1). Also, the number of glutamine residues in the protein (“Protein.GLN), the number of histidine residues in the molecular neighborhood with respect to barycenter (“bar.HIS”), and the pH has a high impact. The first three features may correlate with one another since both glutamine and histidine residues have N atoms in their side chains.

While it is not always straightforward to rationalize the importance of these features, in the following, we provide a reasonable explanation of the importance of some of them, on the basis of previous studies.

A lower number of N atoms in the side chains of residues around the cofactor leads to a smaller *E*_m_. Average values of the feature have a negligible impact on the output, whereas high values tend to be associated with higher *E*_m_ values. Amino acids that have atoms of N are lysine (Lys), arginine (Arg), histidine (His), and tryptophan (Trp). With the exception of Trp, all the above-mentioned amino acids can be protonated, influencing the charge of the molecular neighborhood. Since the higher the positive charge of the flavin environment is, the easier is the flavin reduction, it is reasonable that the increase in Lys, Arg, and His numbers in proximity to the flavin cause a shift of the redox potential toward more positive values. This is also in line with several investigations on flavoproteins, such as flavodoxins, revealing that the flavin redox potential can be tuned by controlling the number of charged residues around the cofactor.^16,19,61^ To corroborate this fact, we analyzed the Spearman correlation between “nats in side chain” and all the other features (Supporting Information File S2), and it turned out that this feature has a high correlation with the number of arginine residues (*r* = 0.872) and of positive residues (*r* = 0.871) in the flavin surrounding.

It can be noticed (Figure 4b) that most of the flavoprotein entries have an experimental pH value ∼ 7, which corresponds to the average value. Therefore, as expected, the violin plot shows that the impact tends to zero. However, high values (alkaline pH, in red) and low values (acid pH, in blue), although few compared to the average values, tend to be selectively localized to the left and right of the plot, respectively. On the whole, it is therefore possible to affirm that an acid pH corresponds to higher *E*_m_, while an alkaline pH results in lower *E*_m_. Such a correlation can be explained by referring once again to the charge of the flavin surrounding: high concentrations of protons lead to positively charged amino acids and consequently to higher *E*_m_.

Also, the hydrophobicities of the residues around N5 (Around_N5.Hydrophobicity) influence the redox potentials: the greater the hydrophobicity is, the more unfavorable the flavin reduction becomes. Since the N5 of the flavin gets protonated upon the first or second reduction (Scheme 1), the reduced forms of the isoalloxazine should be destabilized by a highly hydrophobic neighborhood of N5, which would lower the cofactor redox potential.

Finally, it is interesting to note how a high number of residues capable of forming hydrogen bonds in the protein correlates with higher redox potentials. The content of H-bonds in a protein can be related to its polarity, so a higher number of H-bonds corresponds to a higher polarity of the protein and thus to a higher redox potential value. Furthermore, residues accepting/donating H-bonds found in close proximity to the flavin may also be involved in direct interactions with the isoalloxazine ring. This would cause a shift of redox potential in the positive direction by lowering flavin’s lowest unoccupied molecular orbital energy levels, as indicated previously.^23,62−66^

## Conclusions

The accuracies of ML-based QSPR models strictly depend on the quality of the training data. Labeling a proper number of training samples requires an extensive search and manual curation of experiments reported in the literature. To reduce the heterogeneity of the population to be sampled, we focused at first instance on a single family of proteins. Specifically, we selected the family of flavoproteins because of their relevance in redox processes and the consequent major interest in the prediction of their redox potential. We proved the possibility of using a ML-based QSPR model to predict the redox potential of flavoproteins.

Among the various ML estimators tested in our QSPR analysis, XGB demonstrated superior ability in terms of MAE, RMSE, R2, and Spearman metrics. This result is consistent with recent work that suggests that in general tree-based models perform very well for tabular data.^67^ The reduction potentials predicted with our ML approach are characterized by an average error of ∼36 mV, which is comparable to or even better than that obtained using more sophisticated (and therefore time-consuming) computational methods.^33,34,68^ Indeed, an error of less than 1 kcal/mol was also obtained by Sattelle and Sutcliffe who used thermodynamic integration to quantify the redox potential variation of long-chain *Anabaena* flavodoxin upon site-specific mutations.^34^ The MAE values that we obtained for some specific objects (i.e., wild type *Clostridium beijerinckii* flavodoxin and its G57D and G57T mutants) are also comparable to the ones reported in the literature, obtained with computations based on the electrostatic continuum model by solving the linear Poisson–Boltzmann equation (15.6 and 16.0 mV, respectively).^61^

In addition, it was possible to rationalize, on the basis of previous observations and considerations, both the weight and nature of some of the molecular descriptors that have high impacts on the prediction. Remarkably, our approach also highlighted a series of other protein properties that can influence redox potential, although less intuitively. This information could turn useful in protein engineering applications, aiming at quantitatively tuning flavoprotein redox potential by targeted sequence modifications. In the absence of the experimental 3D structure of a flavoprotein and/or its mutants, the prediction may be extended also to computationally derived models (if characterized by high confidence) that can be obtained via homology modeling, *in silico* mutagenesis or *ab initio* structure prediction using, for instance, AlphaFold.^69^

The performance of the described ML-based QSPR model will benefit from future collection of new experimental data, allowing a further increase in the homogeneity of the flavoprotein set, especially in regions of slightly negative and/or positive potentials. An increase of experimental information would also allow the development of predictive models for *E*_1_ and *E*_2_ or for the gap between the two. This last application would be particularly intriguing, since the separation between flavin first and second redox potentials has recently emerged as a key feature for the design of electron bifurcating proteins, with potential implications in the context of energy conversion.^54,58,70−73^ Finally, an important advantage of the model is that all the considered features can be automatically extracted from the PDB file of the associated protein. We would also like to specify that we reperformed predictions including additional features, describing the pattern of interaction of the isoalloxazine ring of the flavin with the protein neighborhood (e.g., presence of H-bonds or aromatic/aliphatic stacking interactions between one atom of the isoalloxazine ring and the protein matrix). However, none of these features had a significant impact on the prediction. Furthermore, the inclusion of such information did not increase the performance of our estimators (data not shown), suggesting that our chosen descriptors, that are exclusively based on the protein atomic coordinates, implicitly account for it. Such features are independent from the chemical nature of the cofactor, so the same framework could be applied to other families of proteins to determine their redox potentials. Only when training data will be available for other families of proteins we will be able to test whether our model can be generalized to different families of proteins, indicating that general principles were uncovered or, on the contrary, whether ML models need to be trained specifically for each family.

The encouraging results that we obtained for flavoproteins pave the way for a community effort to collect training datasets for other families of proteins.

## Methods

### ML Models

As regression models, we considered the linear regression (LR), Gaussian process regression (GPR),^74,75^ support vector regression (SVR),^76^ k-nearest neighbors regression (KNR),^77^ and two decision tree ensemble methods, random forest (RF)^78^ and gradient boosting (GB).^79,80^ A detailed description of these methods is available in Supporting Information File S1.

### Performance Metrics

All the ML models are evaluated on the basis of different evaluation criteria. The main evaluation criterion used for hyperparameter selection in this paper is mean absolute error (MAE). The smaller the values of MAE are, the higher are the performances of the model. To compare different models, we used other evaluation metrics, namely root mean squared error (RMSE), square correlation coefficient (R2), and Spearman correlation (SC).

### Dataset Reconstruction

The dataset that we used to develop the ML models consists of 141 records, i.e., experimental studies in which midpoint redox potential (*E*_m_) of a flavoprotein was measured. Some of these records have been obtained from the Flavoprotein Database (http://flavoproteindbwebdev-theflavoproteindatabase.webplatformsunpublished.umich.edu/), whereas the other records were collected by us by applying a systematic literature search strategy. In more detail, such a strategy consisted of two phases. First, we searched the Protein Data Bank (PDB) for 3D structures belonging to the flavoprotein class and containing FAD or FMN as cofactors. Then, for each of these, we searched on Scopus for possible works in which the redox potential was measured, using as keywords the name of the flavoprotein, the organism from which the 3D structure was isolated and purified, and the term “mV” (i.e., milliVolt). If different PDBs are available for the same flavoproteins, we selected the one for which there is the most similarity between the experimental condition used to obtain the crystallographic structure and to measure the redox potential. If the *E*_m_ of a flavoprotein was measured at a different pH, we selected and included all these records.

The correspondence between flavoprotein and flavin is not bijective, since there are many flavoproteins containing more than one flavin cofactor. To manage this fact, we applied the following procedure:

- When the flavoprotein has just one flavin cofactor (FMN or FAD), we report in the dataset the molecular descriptors of the interaction between the cofactor and the associated chain of the flavoprotein.
- When the flavoprotein has two or more flavin cofactors, we report in the dataset one example, where the values of the molecular descriptors correspond to the mean over the various chains containing a flavin cofactor.

### ML Experimental Setup

To perform both hyperparameter optimization and model selection, we used a nested cross validation. We used a 5-fold cross-validation procedure for model hyperparameter optimization nested inside a 10-fold cross-validation procedure for model selection. The 5-fold cross-validation procedure involves fitting a model on all folds but one and evaluating the fit model on the holdout fold (i.e., validation set). Under this procedure, the hyperparameter search does not have the opportunity to overfit the dataset as it is only exposed to a subset of the dataset provided by the outer cross-validation procedure. For each estimator, the hyperparameters were selected as the ones which minimize the MAE scores using a grid-search strategy. We repeated this procedure 10 times to explore the feature space extensively and evaluate performance variability, avoiding possible bias due to the stochasticity of the split procedure.

We applied several preprocessing operations on the dataset. First, since the pH values at which the measure of redox potential was carried out are not always present in the corresponding literature, i.e., is a missing value, we replaced it with the mean value computed on the training set. Then, we removed all the features having no variance or having high correlation (Pearson correlation above 0.99) with other features in the training set.

When LR, SVR, KNR, or GPR are considered as ML models, we standardize the features by removing the mean and scaling to unit variance, whereas for both RF and XGB, such a preprocessing operation is not necessary.

Given that the number of descriptors exceeds the training data size, we also applied feature selection to reduce the number of descriptors and avoid possible overfitting during the training process. In more detail, for LR, SVR, KNR, and GPR models, we applied an elastic net (EN) strategy to reduce the number of descriptors before the training process. EN is a regression method that obtains a linear model that estimates sparse coefficients, minimizing a specific cost function
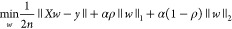
where *n* is the number of training samples, *w* the coefficients of the linear model, α ≥ 0 a constant value which weighs both the *L*^1^ and *L*^2^ regularization terms, and 0 ≤ ρ ≤ 1 a parameter which weighs the two penalty terms. The advantage of such a method is that it allows for learning a sparse model with few of the weights *w*_*i*_. Indeed, trying to minimize the cost function, EN selects those features that are useful, discarding the useless or redundant features, making its coefficient equal to 0. So, the idea of using EN for feature selection consists of using only those features that have coefficients different from 0. Since the choice of α and ρ for EN could strongly affect the results of the feature selection, we tested two different values for α, i.e., α = 10 and 100, and three different values for ρ, i.e., 0.5, 0.75, and 1.

RF and XGBoost models apply feature selection automatically to build their own trees during the training process. However, since too deep of trees can cause overfitting, we selected five as the maximum depths of the trees for both RF and XGB, and we tested three different values (3, 4, and 5) for this hyperparameter.

For hyperparameter optimization, we used a grid-search strategy over a parameter grid. In more detail, we used the grid search provided by GridSearchCV of the Scikit-Learn library,^81^ which generates candidate configurations from a grid of hyperparameter values. The descriptions of the grids, specific for each estimator, are reported in Supporting Information File S2. Any other hyperparameter of the model is set to the default value provided by the original code.

### Interpretation of ML Models Using SHAP

Once a ML model to predict redox potential was obtained, we used SHapley Additive exPlanations (SHAP)^82^ to interpret the result. SHAP is a quite recent methodology that enables quantitative estimation of model interpretability. SHAP uses concepts from cooperative game theory, assigning to each feature a score based on its impact on the model prediction when the feature is present or not during the SHAP estimation. In order to explain complex models, SHAP uses a linear additive feature attribute method as a simpler explanation model
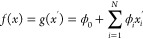
where *f* is the original ML model, *g* the simpler explanation model, *N* the total number of features, *ϕ*_*i*_ the SHAP values measured across all possible inputs, and *x*′_*i*_ the simplified input vector that indicates if a particular feature is present or not during the estimation; ϕ_0_ is associated with the model prediction when all the attributes are not considered in the estimation.

## Data and Software Availability

The code and data used in this work are publicly available at the GitHub repository https://github.com/CompBtBs/Prediction_Flavoprotein_EM. All the PDB structures have been downloaded from the Protein Data Bank (PDB). For all the estimators, except for GB, we used the implementations provided by Scikit-Learn. For GB, we used the XGB implementation provided by Chen and Guestrin.^80^ To analyze the importance of the variables in the XGB model, we used the SHAP (SHapley Additive exPlanations) Python library.

## Supporting Information Available

File S1: A brief description of the machine learning regression models used in this study and correlation plot between the feature “Bar.nNats in side chain” and the observed midpoint potential across the various flavoproteins (PDF)
File S2: Input flavoproteints dataset. Correlation between the feature “Bar.nNats in side chain” and all the other features. P values of Mann–Whitney U rank test. Search ranges for hyperparameter tuning of each considered estimator (XLSX)
