# Supplementary material for: Machine Learning for Efficient Prediction of Protein Redox Potential: The Flavoproteins Case
Source: J Chem Inf Model. 2022 Sep 20;62(19):4748–59. doi: 10.1021/acs.jcim.2c00858 (PMC9554915; doi:10.1021/acs.jcim.2c00858)

SUPPORTING INFORMATION

File S1

Machine learning for efficient prediction of protein redox potential: the flavoproteins case.

Bruno Giovanni Galuzzi[1,3+], Antonio Mirarchi[1+], Edoardo Luca Viganò[2], Luca De Gioia[1], Chiara Damiani[1,3], Federica Arrigoni[1*]*

[1]Department of Biotechnology and Biosciences, University of Milano-Bicocca, Piazza della Scienza 2, 20126, Milan, Italy.

[2] Istituto di Ricerche Farmacologiche Mario Negri, Via Mario Negri 2, 20156 Milan, Italy.

[3] SYSBIO Centre of Systems Biology/ ISBE.IT, Piazza della Scienza 2, 20126, Milan, Italy.

[+]Equally contribution to this work as first authors

[*]Corresponding authors

Index:

- **ML models description.** (S-1 – S-3)
- **Figure S1**: Correlation plot between the feature "Bar.nNats in side chain" and the observed midpoint potential across the various flavoproteins. (S-4)

ML models description.

Linear regression (LR) LR is one of the simplest parametric methods to model the relationship between the input variables (or explanatory variables) and the dependent variable (or response variable). The main assumptions are that there is a linear relationship between the input and output variables, and homoscedasticity, i.e. the residuals (or error terms) must possess a constant variance, regardless of the independent variable. Such a linear relationship is represented in a linear equation as follows:

$$y = w_0 + w_1 x_1 + \ldots + w_n x_n \,,$$

where *n* is the number of input variables, x_i the i-th feature, and w_i the i-th model parameter. The coefficients w_i can be estimated in an efficient way using Least Squares Method.

Gaussian process regression (GPR) GPR, also known as Kriging, can be thought as a generalization of the Gaussian probability distribution to infinitely many variables. A Gaussian process (GP) is a Gaussian random function, and is fully specified by a mean function $m(x)$ and a covariance function $k(x, x')$, as follows:

$$y = GP(m(x), k(x, x')).$$

To define an individual GP, one needs to choose a form for $m(x)$ and $k(x, x')$. In most applications there is no prior knowledge about the mean function, $m(x)$, so, by simplicity, and because GPs are, by definition, a linear combination of random variables with normal distribution, this is commonly assumed to be zero. The covariance function, $k(x, x')$, can be in general any function such that $k(x, x')$ generates a non-negative definite covariance matrix *K*. There are many possible covariance functions, but one that is most frequently used is the squared exponential covariance function, also known as radial basis function (RBF):

$$k(x, x') = \sigma^2 \, exp\left(\frac{1}{2l^2} |x - x'|^2\right).$$

It is easy to see that for this equation, the covariance between any two inputs is really close to one if the inputs are close to each other, and decreases exponentially as the distance between the inputs increases. Here, σ and *l* are the hyperparameters of the kernel which are dataset-dependent and are estimated during the training phase.

Support vector regression (SVR) In the SVR, the purpose is to find a function f(x) that has at most ε deviation from any output y_i for all the training data, and simple as

possible. In its linear formulation, a SVR model is similar to a linear model, but the coefficients w_i are estimated as follows:

$$min\ \frac{1}{2}|w|^2 + C\sum_{i=1}^{l}\ (\xi_i + \xi_i{}^*)$$

subject to

$$y_i - < w, x_i > -b\ \leq \epsilon + \xi_i,$$
$$< w, x_i > +b - y_i\ \leq \epsilon + \xi_i{}^*,$$
$$\xi_i,\ \xi_i{}^* \geq 0,$$

where ξ_i, $\xi_i{}^*$ are slack variables to assure the feasibility of the problem, and $C > 0$ determines the trade-off between the flatness of f and the amount up to which deviations larger than ε are tolerated. To cope with non-linear effect, it is possible to introduce a non-linear kernel function $k(x, x') =< \phi(x), \phi(x') >$, where ϕ is a non-linear map $\phi: X \rightarrow \Omega$ from the original space of the input variables X and a new feature space Ω. In this case, the learning function becomes

$$f(x) = \sum_{i=1}^{l}\ w_i\, k(x_i, x) + b.$$

K-nearest neighbors regression (KNR) KNR belongs to the family of instance-based learning algorithms, where the training instances are stored in memory without explicitly learning a model. The training instances are processed in the prediction phase. For each new data instance, a query is made and, in its simplest case, the query returns the k-nearest data points $\hat{x}_i$ to the new instance, based on some distance or similarity metric. In KNR, the output is determined by averaging the outputs $\hat{y}_i$ of the k-neighboring data points:

$$f(x) = k^{-1}\sum_{i=1}^{k}\ \hat{y}_i\,.$$

Regression Trees. Regression trees are supervised learning models, where the main idea consists of splitting a dataset into smaller subsets while at the same time an associated decision tree is incrementally developed. The final result is a tree with decision nodes and leaf nodes. A decision node has two or more branches, each representing values for the feature tested. Leaf node represents a decision on the numerical target. Usually, a decision tree is built top-down from a root node and involves the partitioning of the data into subsets $R_1, R_2, \ldots, R_T$, that contain instances with similar values, and fitting a simple model in each one. The corresponding regression model becomes:

$$f(x) = \sum_{i=1}^{T}\ c_m\, I(x \in R_m\,),$$

where $I(x) = 1$, if $x \in R_m$ and 0 otherwise, and c_m is the average of y_i in region R_m:

$$c_m = avg(y_i | x_i \in R_m).$$

The partition of the data is usually done iteratively with a greedy algorithm, in which, at each step, the best pair *(j,s)* is chosen, where *j* is the splitting variable, and *s* is the splitting point.

Random forest (RF) is an ensemble learning method, where multiple regression trees are trained in parallel without any interaction between each other. Each tree is usually trained in a different bootstrap version of the original dataset, selecting a subset of variables at random from the original variables. The output of the model results from the average of the predictions of the various trees.

Gradient boosting (GB) GB is an ensemble method, in which multiple regression trees are built. Unlike random forest, the decision trees in gradient boosting are built additively; i.e. each decision tree is built one after another. Each new tree is built to improve the previous one (boosting). The gradient part of GB comes from minimizing the gradient of a loss function, which is used by the algorithm to build each tree.

Figure S1. Correlation plot between the feature “Bar.nNats in side chain” and the observed midpoint redox potential across the various flavoproteins.

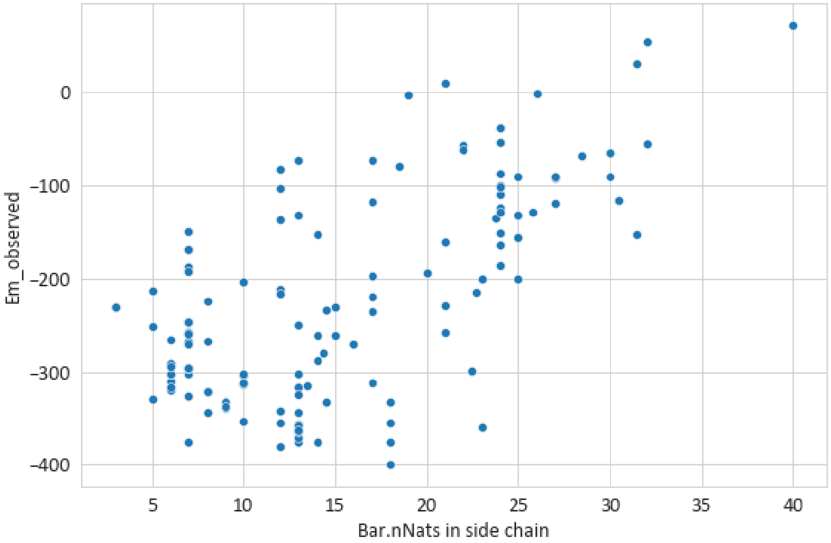

Supplement: Supplementary file 1 — ci2c00858_si_001.pdf [file ci2c00858_si_001.pdf]
